# Saving the horseshoe crab: A synthetic alternative to horseshoe crab blood for endotoxin detection

**DOI:** 10.1371/journal.pbio.2006607

**Published:** 2018-10-12

**Authors:** Tom Maloney, Ryan Phelan, Naira Simmons

**Affiliations:** 1 Revive & Restore, Sausalito, California, United States of America; 2 Wilson Sonsini Goodrich & Rosati, San Francisco, California, United States of America

## Abstract

Horseshoe crabs have been integral to the safe production of vaccines and injectable medications for the past 40 years. The bleeding of live horseshoe crabs, a process that leaves thousands dead annually, is an ecologically unsustainable practice for all four species of horseshoe crab and the shorebirds that rely on their eggs as a primary food source during spring migration. Populations of both horseshoe crabs and shorebirds are in decline. This study confirms the efficacy of recombinant Factor C (rFC), a synthetic alternative that eliminates the need for animal products in endotoxin detection. Furthermore, our findings confirm that the biomedical industry can achieve a 90% reduction in the use of reagents derived from horseshoe crabs by using the synthetic alternative for the testing of water and other common materials used in the manufacturing process. This represents an extraordinary opportunity for the biomedical and pharmaceutical industries to significantly contribute to the conservation of horseshoe crabs and the birds that depend on them.

## Introduction

The 450 million-year-old horseshoe crab has been integral to the safe manufacturing of vaccines, injectable medications, and certain medical devices. Populations of all four extant species of horseshoe crab are in decline across the globe, in part because of their extensive use in biomedical testing [1]. From annual population surveys, it is clear that the biomedical industry’s dependence on the horseshoe crab in North America is ecologically unsustainable. A synthetic alternative has been commercially available for more than a decade, and it has been unclear why the pharmaceutical industry continues to rely on the horseshoe crab. To answer this question, Revive & Restore, a nonprofit organization focused on protecting endangered species, researched the industry and interviewed industry experts [1].

In this paper, we synthesize 10 studies validating the efficacy of the synthetic alternative. Our review dispels lingering misconceptions and highlights the opportunity for the pharmaceutical industry to immediately embrace a new detection technology for common manufacturing materials, which will reduce the need to bleed horseshoe crabs by 90%. Phased adoption will build the empirical data to confirm that synthetic endotoxin detection methods can be safely implemented, ending the industry’s dependence on animal-based technologies. This transition is a critical step in turning the tide for the horseshoe crab and for the migratory birds that rely upon them.

## Importance of the horseshoe crab to the pharmaceutical industry

In the United States, every drug approved by the US Food and Drug Administration (FDA) must be tested for bacterial contaminants. Endotoxins, common and potentially dangerous contaminants present in the outer membrane of the cell wall of gram-negative bacteria, can cause life-threatening fever or toxic shock if introduced intravenously, making their detection an essential safety test for the safe manufacture of all injectable medications [2].

For 40 years, from the 1940s to the 1970s, the pharmaceutical industry relied on rabbits to detect endotoxins, and hundreds of thousands of rabbits were euthanized annually. But by 1970, a new technique for endotoxin detection had been developed, using the blood of the horseshoe crab. It had been discovered that the horseshoe crab had a primitive but highly sensitive immune response to endotoxin contamination [3]. This primitive defense system is expressed in amebocyte cells, which circulate through the horseshoe crab hemolymph. The amebocytes are extremely sensitive to the lipopolysaccharide (LPS) found in endotoxins. When hemolymph comes into contact with gram-negative bacteria or LPS, the amebocytes begin to degranulate, and hemolymph coagulation is initiated by the granule components [4]. The reaction between the amebocyte and bacterial contaminants is the basis of the Limulus amebocyte lysate (LAL) test—the current standard for endotoxin testing around the world [5,6]. When implemented 40 years ago, the transition to the more efficacious LAL represented an incremental step away from laboratory animal-based endotoxin detection technologies, relying instead on blood from wild horseshoe crabs. Today, all injectable medications—as well as some environmental samples and medical devices—are screened for endotoxin contamination using the LAL test.

**Fig 1:**
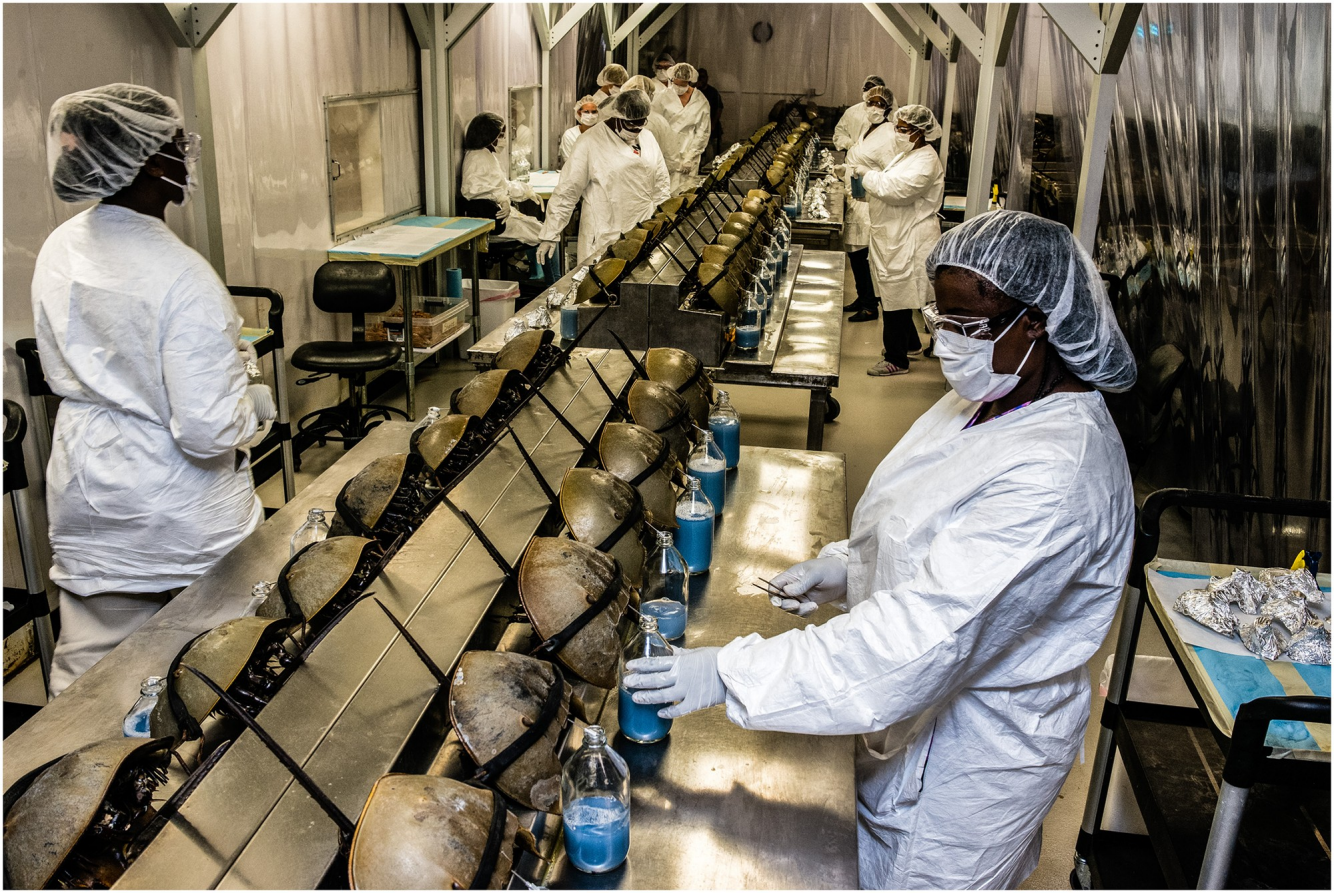
Horseshoe crabs are bled at the Charles River Laboratory in Charleston, South Carolina, US. Photograph by Timothy Fadek.

To create the LAL test, horseshoe crabs are captured and bled **(**Fig 1). The blood cells are then centrifuged and lysed in distilled water to release the cascade of enzymes responsible for recognizing endotoxins. To test a sample for endotoxins, a sample is mixed with the lysate at a specified ratio. Generally, the product of this reaction is detected as a gel clot, but it can also be successfully detected with chromogenic and turbidimetric techniques [7].

The horseshoe crab–derived test can be made from any one of the four extant species of horseshoe crab: *Tachypleus tridentatus* in Asia, *T*. *gigas* in Southeast Asia, *Carcinoscorpius rotundicauda* in Southeast Asia, and *Limulus polyphemus* in the Atlantic Ocean off North America [8].

## Ecological impact of biomedical bleeding

Each year on the East Coast of the US, the biomedical industry captures approximately 500,000 horseshoe crabs and drains as much as a third of their blood. On average, 13% of the bled crabs are sold as bait for other fisheries, according to the Atlantic States Marine Fisheries Commission, while the rest are returned to the ocean [9]. Because horseshoe crabs are aquatic animals, the time out of water, combined with the bleeding process itself, is a significant source of injury and potential mortality. Conservation groups estimate the mortality rate of released crabs to be at least 15% to 30%. Regulators of regional fisheries use the 15% figure to guide management. Between the crabs sold for bait after bleeding and conservative estimates of mortality resulting from bleeding, at least 130,000 horseshoe crabs are killed annually by the biomedical industry.

Multiple studies have shown that during the weeks following bleeding, horseshoe crabs also experience detectable sublethal effects such as injury and disorientation [10,11], which lead to increased incidence of disease and possibility to lower spawning rates. The long-term effects of the bleeding procedure on breeding fitness are not yet understood.

## Characterization of the LAL test derived from horseshoe crabs

The importance of endotoxin detection lead to an extensive characterization of the enzymatic components in the LAL endotoxin recognition cascade [12]. The LAL cascade is based on three kinds of serine protease zymogens—factor C, factor B, and proclotting enzyme z—plus coagulogen, a clottable protein. Endotoxins, notably LPS, activate the zymogen factor C to the active form, factor C [13,14,15]. Factor C then activates factor B to active factor B, which in turn converts the proclotting enzyme to the clotting enzyme. Each activation proceeds by limited proteolysis. The resulting clotting enzyme cleaves two bonds in coagulogen, which is a fibrinogen-like molecule in arthropods such as the horseshoe crab, to yield an insoluble coagulin gel [16]. The first molecule in the cascade, factor C, is the key molecule responsible for initiating the coagulation cascade system in the horseshoe crab hemolymph.

## The development of a synthetic alternative

In 1997, scientists at the National University of Singapore, Ling Ding Jeak and Bo How, realized the potential that cloned, laboratory-synthesized recombinant Factor C (rFC) could have for the development of an animal-free endotoxin detection technology. They were the first to clone the DNA of a factor C molecule and synthesized rFC, the synthetic alternative to the LAL test [17,18]. In contrast to LAL, the synthetic alternative utilizes a single protein cloned from a horseshoe crab as its active ingredient [19]. Subsequently, other groups cloned factor C molecules from different horseshoe crab species and studied them carefully to develop various rFC tests [8,20,21,22,23]. In the rFC test, the binding of endotoxin activates the synthetic rFC molecule, which then cleaves a fluorogenic substrate, resulting in the generation of a fluorogenic compound. The fluorescence is measured twice, first at time zero and then after the endotoxin has been introduced. The difference in fluorescence is proportional to an endotoxin concentration in the sample and is used to calculate a final endotoxin result.

Despite the optimism that the original development of rFC and similar recombinant technologies would largely displace the use of LAL, this did not occur. One commonly cited reason for the poor adoption was concern over the efficacy of rFC when compared to LAL.

## Resolving questions about efficacy of the synthetic alternative

We reviewed multiple studies that evaluated the efficacy of rFC as an endotoxin detection method, summarized in Table 1. These studies tested a variety of different samples for endotoxin contamination. For the detection of gram-negative bacterial endotoxin, the rFC-based assay proved to be equivalent to the LAL test both in its ability to quantifiably measure endotoxin and in its ability to detect endotoxins across a range of concentrations. Furthermore, rFC is specific to endotoxin detection, whereas LAL is a nonspecific test: peptidoglycan from gram-positive bacteria, exotoxins from group A Streptococci, simple polysaccharides including yeast mannans and bacterial dextrans, and dithiols all activate LAL to give false positive results [24]. Notably, rFC does not contain glucan-sensitive factor G, meaning the rFC-based assay is not subject to false positives like the LAL test is.

**Table 1:** List of reviewed rFC efficacy studies.

Author(s)	Affiliations	Publication
Abate and colleagues [25]	Plymouth University University of Bristol University of the West of England	July 2017
Bolden and Smith [17]	Eli Lilly, Lilly Corporate Center, Indianapolis	July 2017
Schwarz and colleagues [26]	University of Salzburg	Dec 2014
Reich and colleagues [27]	Hyglos GmbH	June 2014
Chen and Mozier [28]	Pfizer, Inc.	March 2013
Grallert and colleagues [29]	Hyglos GmbH	Oct 2011
McKenzie and colleagues [30]	University of Massachusetts Harvard School of Public Health	May 2011
Thorne and colleagues [31]	University of Iowa Colorado State University	August 2010
Loverock and colleagues [18]	Lonza Walkersville, Inc.	Nov 2009
Bolden and colleagues [32]	Eli Lilly, Lilly Corporate Center, Indianapolis Genentech Bayer HealthCare LLC	July 2017

**Abbreviation**: rFC, recombinant Factor C.

Each of these 10 studies demonstrated that commercially available rFC tests detect endotoxins with results equivalent to or better than LAL, regardless of which company manufactured it. The breadth of these studies also showed strong efficacy across a range of uses and demonstrated high sensitivity, strong reliability, and other positive considerations in the clinical use of rFC.

### Sensitivity

The rFC assay demonstrated both a high rate and reliable sensitivity (in picogram quantities) of endotoxin detection for a variety of LPS structures [25]. The study conducted by Abate and colleagues found that, even at low amounts, the synthetic alternatives detected endotoxins with a wide range of structures. This is important because Lipid A from *Escherichia coli* LPS typically has a hexa-acyl structure, while LPS from different gram-negative bacteria may have different numbers and arrangements of acyl chains [25]. This is the first study to demonstrate that rFC assays can detect these various assay structures. This is significant in terms of reassuring laboratory personnel that rFC products exhibit strong sensitivity.

### Range of applicability

Exposure to endotoxin poses a potential health risk in diverse clinical and nonclinical settings. Therefore, several of the studies listed above sought to confirm the suitability of rFC as an endotoxin assay in a variety of settings. For instance, Reich demonstrated that three commercially available synthetic reagents showed a 94.4% correlation to each other when testing water from a variety of sources, including lakes, springs, tap water, mineral water, and deionized water [27]. Also, a comparison of LAL and rFC for the assessment of airborne endotoxins found that the LAL and rFC assays are similar to air samples drawn from a variety of agricultural environments and over a wide range of concentration [30]. Furthermore, both methods yielded few nondetectable values. In a manufacturing setting, Chen and Mozier tested 13 therapeutic protein solutions at various stages of manufacturing and confirmed Schwarz’s findings regarding the viability of rFC as a replacement for LAL.

### Reliability

An important outcome of two of the studies above was that rFC demonstrates a higher rate of specificity for endotoxin [17,27]. As previously mentioned, LAL is not specific for endotoxin detection. It is well known that LAL testing cross-reacts with several β-glucans. Common sources of glucans include fungi (or yeast hydrolysate). Other sources, also ubiquitous in pharmaceutical manufacturing, are filters and other products made from cellulose materials, plant-derived raw materials, cotton-containing enclosures, sugars, and other naturally derived raw materials.

In addition to the issues caused by the presence of β-glucans, false positives can also be caused by proteases or phospholipids. There are no known false positives currently reported for rFC-based tests. Furthermore, buffers or solvents have been known to inhibit the sensitivity of LAL resulting in potential false negatives. Grallert and colleagues found that rFC overcame other sources of unreliable results that occur during LAL testing, including inhibitory constituents of the sample; fewer invalid results, which necessitate retesting; less interference in complex samples; and a broad dynamic range of 0.05 endotoxin units per milliliter (EU/ml) to 500 EU/ml [29].

Another reliability factor to consider is the lot-to-lot variability of the reagent used to detect endotoxin. One study that tested four different extraction and assay media concluded that on issues of lot-to-lot variability, the results of the rFC assay kits were superior to those previously reported for LAL assays [29]. Any variability encountered was easily overcome by using a standardized protocol for each test.

### BioPhorum Operations Group study

In perhaps the strongest study, the BioPhorum Operations Group (BPOG), an industry consortium of biopharmaceutical manufacturers, formed a workgroup to develop a harmonized study design for assessment of endotoxin recovery with LAL, rFC, or control tests. Fourteen biopharmaceutical manufacturers, including Amgen, AstraZeneca MedImmune, Bayer, Biogen, Bristol-Myers Squibb, Eli Lilly, Johnson & Johnson Janssen, Lonza, Merck & Co. (USA), Merck Serono, Regeneron, Roche/Roche Genentech, and Sanofi/Sanofi Genzyme, performed experiments using similar protocols to determine if multiple laboratories would reach similar conclusions in detecting endotoxins with both LAL and rFC reagents. The study (hereinafter BPOG study) was jointly authored by laboratory heads at Eli Lilly, Genentech, and Bayer. They describe a robust, large-scale evaluation of both reagent types under different conditions, with tests conducted in 21 different biopharmaceutical laboratories [32]. In total, the study compared 37 different LAL/rFC reagent and supplier combinations. The study evaluated the three principal LAL test methods: the gel-clot, turbidimetric, and chromogenic methods. The study also evaluated the performance of rFC against the legacy LAL diagnostics.

The results of the BPOG study are striking. Although some variability was observed between tests, the BPOG study demonstrated that rFC can successfully detect naturally occurring endotoxins with a high limit of detection and in the presence of test “inhibitors,” such as sodium citrate buffers.

Importantly, these data suggest that rFC is at least comparable, if not better than LAL in detecting endotoxins under various buffer conditions. The BPOG study further outlined a harmonized protocol that yielded consistent results across many different laboratories, regardless of whether the test was conducted with naturally occurring endotoxins or purified LPS. Importantly, the BPOG study demonstrated that effective and consistent results can be obtained with existing commercially available rFC reagents.

## Removing barriers to the adoption of rFC

Although there is now abundant evidence that the efficacy of rFC is equivalent to or better than LAL in the detection of endotoxin, adoption of new technology is difficult, and change has come slow to the industry. The rFC assay has been commercially available since 2003, yet the pharmaceutical industry has been hesitant to utilize the synthetic alternative for a number of reasons. Endotoxin is a serious health concern; manufacturers and regulators have been justifiably cautious in the adoption of new detection technologies. Because endotoxin testing is highly regulated, pharmaceutical manufacturers have been inclined to follow known methods even when there is an opportunity to innovate.

Because vaccines and drugs are manufactured and distributed worldwide, different regulatory bodies (e.g., US FDA) rely on various compendia (e.g., US Pharmacopeia) and, where possible, a harmonization process to assure uniformity in endotoxin testing methods across all regulatory jurisdictions. In 2012, the FDA issued separate guidance acknowledging the use of rFC as an acceptable alternative to LAL, and the European health ministry followed. But because the use of rFC testing methods have not been incorporated into the harmonized Pharmacopeias, manufacturers must go through the extra step of validating the rFC assay, which is a more burdensome process than the streamlined method of verification used for methods described in the general Pharmacopoeia.

Leadership from the pharmaceutical industry that demonstrates a willingness to modernize laboratory processes and to covert to rFC is essential. Just by converting the testing of water and other common manufacturing materials, 90% of the demand for LAL in large-scale pharmaceutical manufacturing could be displaced, according to endotoxin experts with decades of experience. There is a regulatory distinction between in-line processing and the final testing of the marketable drug product. Most manufacturers have the discretion to convert the testing of common in-line processing materials, such as pharmaceutical-grade water, without an onerous regulatory change process. Furthermore, increased utilization would advance the inclusion of rFC into the harmonized Pharmacopeias and would encourage other pharmaceutical companies to do the same. Until this year, rFC was under an exclusive patent, and pharmaceutical companies were reluctant to rely on a sole rFC supplier for such an important step in the manufacturing process. Regulators too were concerned about endorsing a method only available from a single manufacturer. Today, there are multiple suppliers, and more are expected to enter the market. Pricing is competitive with horseshoe crab-derived products and is likely to become even more advantageous with increased competition pricing resulting from new suppliers entering the field.

## Horseshoe crabs are essential to a healthy ecosystem

In North America, compounding the threats of biomedical bleeding are fisheries pressures and the effects of climate change and rising sea levels, which are diminishing the availability of suitable spawning sites. The current overexploitation of horseshoe crabs is not dissimilar to other mismanaged species that have been driven to extinction. In 2016, the International Union for the Conservation of Nature [33] moved the mid-Atlantic populations of the American horseshoe from “near threatened” to “vulnerable” on its red list assessment. This reinforces the urgency for the biomedical industry to do its part to abate a major threat to the species by adopting synthetic alternatives and ending the use of LAL.

In the mid-Atlantic region of North America, the overharvest of the horseshoe crab is causing significant ecosystem-level impacts. Six species of shorebirds synchronize their northward migration along the Atlantic flyway to gorge on the eggs of spawning horseshoe crabs in Delaware Bay, a critical food stop on their journey to Arctic nesting grounds. Recent research has demonstrated that the abundance of horseshoe crab eggs is vital to both the survival and successful breeding of the birds that rely on them, particularly the red knot (*Calidris canutus rufa*), whose 9,500-mile migration from the tip of South America to the Arctic is among the longest of any bird in the world [34,35,36]. In 2014, a dwindling horseshoe crab population in North America prompted the classification of the red knot as threatened under the US Endangered Species Act [37]. The long-distance migratory birds that depend upon horseshoe crab spawning are some of the most rapidly declining shorebirds in North America.

## Conclusion

The proven efficacy of the recombinant alternative for endotoxin detection provides an opportunity for the pharmaceutical industry to modernize procedures and contribute significantly to the conservation of horseshoe crabs. The move from rabbits to crabs occurred in the late 1970s; it is now time for the industry to modernize its methods and embrace a more humane and ecologically sustainable method of endotoxin testing. Immediate conversion to rFC for the testing of water and other common manufacturing materials presents no risk of diminution in reliability or sensitivity in endotoxin detection and is enabled under current regulatory guidance. Furthermore, based on interviews with industry experts, rFC presents advantages to LAL beyond the urgently needed benefits to conservation. Use of rFC largely eliminates the occurrence of false positive reactions to glucans and other commonly encountered substances. The reagent is more consistent since it is not subject to the lot-to-lot variability found in LAL. Importantly, industry experts have confirmed that conversion to rFC presents potential cost savings, and these are expected to become more significant now that patent protections have expired and more rFC manufacturers are expected to enter the market.

Conversion to rFC would result in a 90% reduction in the demand for LAL, which means that mortality resulting from bleeding would decrease by an estimated 100,000 horseshoe crabs annually in North America alone. The relative threat abatement of widespread conversion to rFC for the three species of Asian horseshoe crab is hard to quantify, but any threat reduction will be beneficial for these species.

Horseshoe crabs face multiple threats, and the need for global conservation provides a remarkable opportunity for the biomedical industry to contribute significantly to their conservation. Given the equivalent efficacy, proven reliability, a clearly defined regulatory pathway, and the profound ecological benefits of ending the bleeding of horseshoe crabs, the authors recommend rapid proactive adoption of the recombinant-based alternatives as the standard method for endotoxin testing in pharmaceutical and biomedical laboratories worldwide.

## Abbreviations

BPOG: BioPhorum Operations Group
LAL: Limulus amebocyte lysate
LPS: lipopolysaccharide
rFC: recombinant Factor C
